# Case report: dose-dependent interaction between dexamethasone and voriconazole in severely ill patients with non-Hodgkin’s lymphoma being treated for invasive pulmonary aspergillosis

**DOI:** 10.3389/fphar.2024.1403966

**Published:** 2024-06-27

**Authors:** Jingjing Huang, Yang Chen, Ming Zhong, Ruoming Tan

**Affiliations:** ^1^ Department of Pharmacy, Ruijin Hospital, Shanghai Jiao Tong University School of Medicine, Shanghai, China; ^2^ Department of Critical Care Medicine, Ruijin Hospital, Shanghai Jiao Tong University School of Medicine, Shanghai, China

**Keywords:** voriconazole trough concentration, dexamethasone dose, drug interaction, case report, therapeutic drug monitoring, aspergillosis, hematological malignancies

## Abstract

**Background:**

Voriconazole is primarily metabolized by CYP2C19 and CYP3A4. Drug interactions that affect this pathway can alter its plasma exposures, resulting in untargeted voriconazole concentrations.

**Case summary:**

In this case report, we describe the case of a 64-year-old man who was treated for non-Hodgkin’s lymphoma with continuous glucocorticoids co-administrated with voriconazole against invasive pulmonary aspergillosis. A decrease in trough concentration (C_min_) of voriconazole was observed and related with co-administration of dexamethasone in the patient carrying the CYP2C19 *1*2 genotype: voriconazole C_min_/dose ratios of 0.018 (0.1 mg L^−1^/5.7 mg kg^−1^ day^−1^), 0.18 (1 mg L^−1^/5.7 mg kg^−1^ day^−1^), and 0.23 (2 mg L^−1^/8.6 mg kg^−1^ day^−1^) at dexamethasone doses of 20, 12.5, and 2.5 mg, respectively. Sub-therapeutic voriconazole C_min_ was associated with high- and moderate-dose dexamethasone (20 and 12.5 mg), leading to failure of antifungal treatment.

**Conclusion:**

The extent of voriconazole–dexamethasone interaction was determined by the dose of dexamethasone and associated with the *CYP2C19* *1*2 genotype. Therapeutic drug monitoring of voriconazole is necessary to avoid clinically relevant interactions for optimal antifungal therapy.

## Graphical Abstract

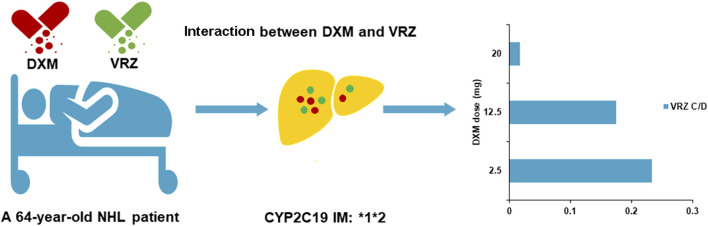

## Introduction

Invasive aspergillosis increases with the increase in the number of immunosuppressed patients, including those with malignant tumors, acquired immune deficiency syndrome, and who have underwent organ transplantation. Infection-related mortality remains high, particularly in severely ill patients with immunologic defects that are irreversible (Sanguinetti et al., 2019; Cadena et al., 2021). Furthermore, recent pandemics such as COVID-19 and influenza increase the morbidity and mortality caused by this invasive fungal infection (Lamoth et al., 2021). Voriconazole is the first-line therapy and widely used to prevent and treat invasive aspergillosis infection (Ullmann et al., 2018). Its therapeutic window (trough concentration, C_min_) is narrow (Wang et al., 2022). Subtherapeutic voriconazole C_min_ (1–2 mg L^−1^) was related with treatment failures, while supratherapeutic C_min_ (4.5–6 mg L^−1^) led to some voriconazole toxicities (Jin et al., 2016). Voriconazole C_min_ exhibits great individual variability. Glucocorticoid–voriconazole interaction and *CYP2C19* polymorphism were important factors that led to intra- and inter-individual variability of voriconazole C_min_ (Moriyama et al., 2017). Dexamethasone, as the most prescribed glucocorticoid, is widely used in chemotherapy regimens for treatment of non-Hodgkin’s lymphoma. However, a potential interaction between dexamethasone and voriconazole can have a clinical impact in immunosuppressed patients carrying *CYP2C19* *2 and *3 alleles. Furthermore, the Asian population has high frequencies of *CYP2C19* mutant genes, including *2 (32%) and *3 (6%–10%) (Mizutani, 2003).

Herein, we reported an adverse dose-dependent interaction between voriconazole and dexamethasone in a non-Hodgkin’s lymphoma patient who was a CYP2C19 carrying intermediate metabolizer (IM, *1*2) and suffered from invasive pulmonary aspergillosis.

### Case description

A 64-year-old Chinese man weighing 70 kg suffering for more than 1 month due to recurrent fever was transferred from an outside medical facility to the Department of Critical Care Medicine, Ruijin Hospital, Shanghai Jiao Tong University School of Medicine for further treatment on 26 May 2022. He was diagnosed with hemophagocytic lymphohistiocytosis (HLH) based on splenomegaly, hemocytopenia, elevated ferritin, hypertriglyceridemia, and abnormal coagulation function, according to the criteria of HLH-2004 (Henter et al., 2007). Then, non-Hodgkin’s lymphoma had been diagnosed through bone marrow biopsy, causing HLH. This patient received continuous glucocorticoid treatment with chemotherapeutic agents, zanubrutinib and obinutuzumab, for the treatment of HLH and non-Hodgkin’s lymphoma (Table 1). He subsequently developed hospital-acquired pneumonia. Carbapenem-resistant *Klebsiella pneumoniae* (CRKP), *Aspergillus fumigatus*, and *Enterococcus faecium* were identified in sputum and blood by metagenomics next-generation sequencing (mNGS) and culture, necessitating initiation of antimicrobial treatment. Antibacterial therapy included empirical piperacillin–tazobactam, polymyxin B, fosfomycin, minocycline, ceftazidime–avibactam, and vancomycin. Voriconazole and amphotericin B were administrated for aspergillosis infection.

**TABLE 1:** Medication list of the patient.

Medication	Date							
	May 26th to June 1st	June 2nd and 3rd	June 4th to 7th	June 8th to 11th	June 12th to 17th	June 18th and 19th (mg)	June 20th to 22nd (mg)	June 23rd to 29th
Treatment of HLH and lymphoma								
					Lymphoma was diagnosed on June 13th			
Dexamethasone (IV)	20 mg	10 mg	5 mg	2.5 mg				
Etoposide (IV)	0.15 g							
Methylprednisolone (IV)					80 mg	60 mg	40 mg	20 mg; 10 mg (June 28th and 29th)
Zanubrutinib (oral)					80 mg bid (↑ June 13th)			
Obinutuzumab (IV)					1000 mg (used on June 16th)			
IVIG			20 g		20 g; 10 g (reduced on June 16th), and 5 g (reduced on June 22nd)			
Antimicrobial therapy								
	CRKP (sputum culture; blood mNGS); *Candida* *albicans* (mid-stream urine culture) on June 1st. Aspergillosis and *Enterococcus* *faecium* (blood mNGS) on June 7th. CRKP, aspergillosis, and *Enterococcus faecalis* (sputum culture; blood mNGS) on June 9th Aspergillosis (sputum mNGS) on June 29th.							
Piperacillin–tazobactam (IV)	4.5 g q8 h							4.5 g q8 h (↑ June 28th)
Polymyxin B (IV)	1.5 mu q12 h(↑June 1st)		1mu q12 h; 0.5 mu q12 h (adjusted on June 7th)					
Polymyxin B (inhale)	0.25 mu q12 h (↑ June 1st)							
Fosfomycin (IV)	4 g q8 h (↑ June 1st and ↓ June 5th)							
Minocycline (oral)		100 mg bid (↑ June 2nd)						
Ceftazidime–avibactam (IV)					1.25 g q8 h (↓ June 25th)			
Vancomycin (IV)			500 mg qd (↑June 7th and ↓ June 25th)					
Fluconazole (IV)	400 mg once on June 1st and 200 mg q12 h (↓ June 4th)							
Caspofungin (IV)			50 mg qd (↑ June 7th and ↓ June 9th)					70 mg once (used on June 29th)
Voriconazole (IV)			400 mg two dose on June 7th; 300 mg q12 h; and 200 mg q12 h (reduced on June 17th)					
Ganciclovir (IV)				0.125 g qd				

Medication	Date								
	June 30th	July 1st to 4th	July 5th to 11th	July 12th to 20th	July 21st to 31st (mg)	August 1st to 4th		August 5th to 11th	August 12th to 18th (mg)
Treatment of HLH and lymphoma									
Dexamethasone (IV)		20 mg			2.5 mg	12.5 mg		2.5 mg	2.5 mg
Treatment of HLH and lymphoma									
Vincristine (IV)		0.5 mg				0.5 mg			
Doxorubicin (IV)		18 mg				18 mg			
Methylprednisolone (IV)	80 mg		10 mg	10 mg					
Zanubrutinib (oral)	80 mg bid								
Obinutuzumab (IV)	1000 mg								
IVIG	5 g	20 g	10 g; 5 g(reduced on July 13th and ↓ July 26th)					10 g (↑August 8th); 5 g(reduced on August 16th)	
Antimicrobial therapy							Pulmonary aspergillosis improved showed by CT scan on August 16th		
			Aspergillosis (sputum mNGS) and a nodule in the right upper lobe and a thick-walled cavity inside the nodule shown by CT scan on July 5th; aspergillosis (blood mNGS; BALF mNGS); CRKP (BALF mNGS) on July 6th; CRKP, aspergillosis, and *Stenotrophomonas maltophilia* (BALF mNGS) on July 15th						
Piperacillin–tazobactam (IV)	4.5 g q8 h (↓ July 14th)								
Minocycline (oral)	100 mg q12 h (↓July 2nd)								
Amikacin (inhale)			200 mg q12 h (↑ July 11th and ↓ July 17 th)						
Ceftazidime–avibactam (IV)						2.5 g q8 h (↑ July 16th)			
Caspofungin (IV)	50 mg (↓ July 24 th)								
Voriconazole (IV)	200 mg q12 h		400 mg q12 h	300 mg q12 h		200 mg q12 h		400 mg q12 h	300 mg q12 h
L-AMB (inhale)			5 mg bid (↓ August 15th)						
ABCD (IV)					200 mg				
Ganciclovir (IV)	0.125 g qd								

ABCD, amphotericin B cholesteryl sulfate complex; BALF, bronchoalveolar lavage fluid; CRKP, carbapenem-resistant *Klebsiella pneumoniae*; HLH, hemophagocytic lymphohistiocytosis; IV, intravenous; IVIG, intravenous immunoglobulin; L-AMB, liposomal amphotericin B; mNGS, metagenomics next-generation sequencing; ↓, medications were started; ↓, medications were stopped.

### Antifungal treatment and dexamethasone dose

**FIGURE 1:**
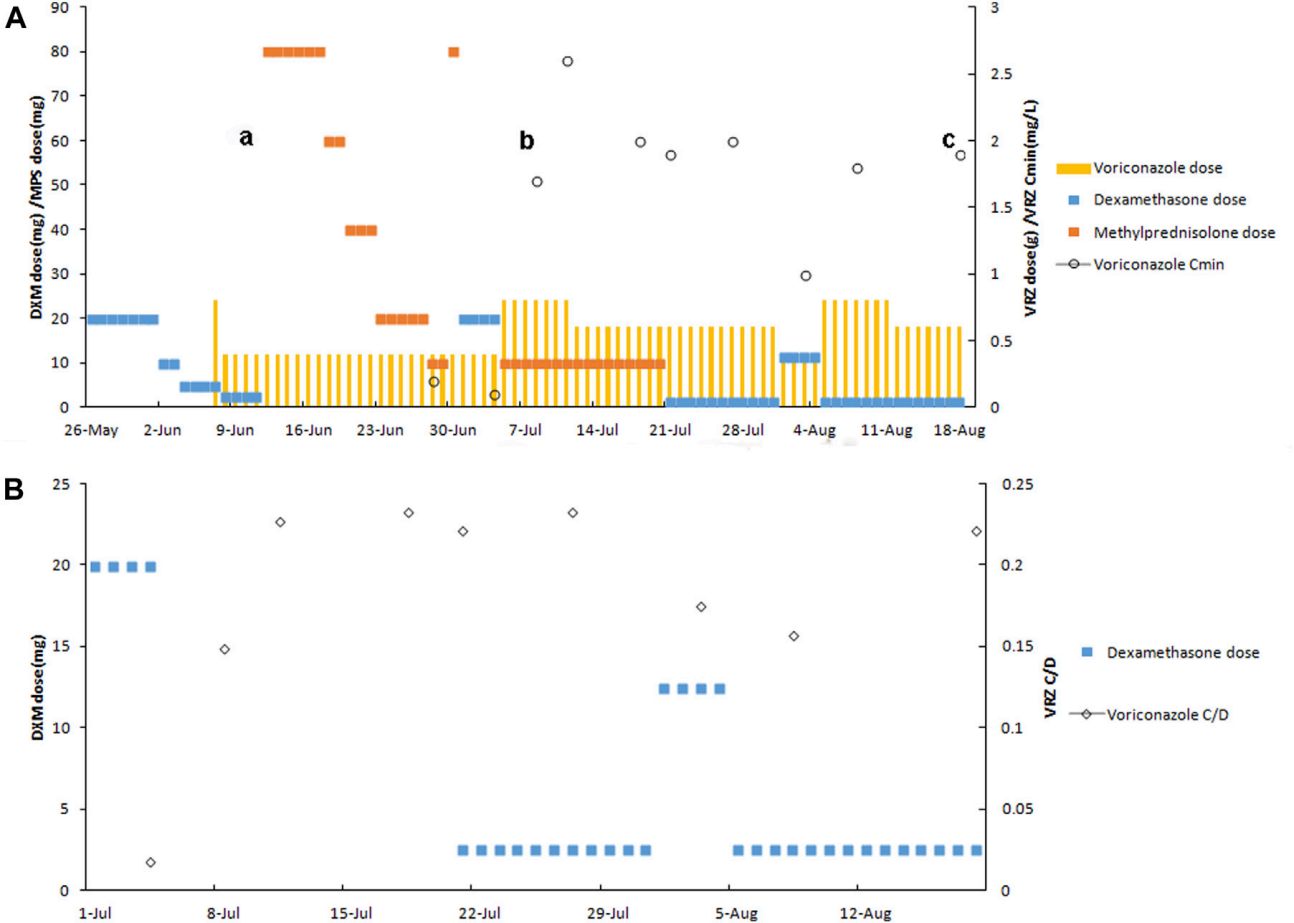
Relationship between the C_min_ **(A)** and C_min_/dose **(B)** ratios of voriconazole and dexamethasone dose. **(a)** *Aspergillus fumigatus* (sputum and blood mNGS). **(b)** Progressive pulmonary aspergillosis with a nodule in the right upper lobe and a thick-walled cavity inside the nodule (chest CT scan) and *Aspergillus fumigatus* (BALF and blood mNGS). **(c)** Pulmonary aspergillosis improved (chest CT scan). Abbreviations: C/D: C_min_/dose ratio; DXM, dexamethasone; MPS, methylprednisolone; VRZ, voriconazole.

The plasma trough concentration (C_min_) of voriconazole was 0.2 mg L^−1^, determined by liquid chromatography–electrospray tandem mass spectrometry (detailed in the Supplementary File S1), on 28 June 2022. Twenty-one days of intravenous (IV) voriconazole (400 mg twice on the first day and maintenance dose of 200 mg q12 h) treatment was not satisfactory (Figure 1). On assessing, *Aspergillus fumigatus* was found again in sputum (mNGS) on 29 June 2022. Before voriconazole initiation, IV dexamethasone 20 mg had been used for 7 days and then gradually reduced. Following dexamethasone, IV methylprednisolone 80 mg was given and then gradually reduced. Subsequently, IV dexamethasone 20 mg combined with etoposide 0.5 mg and vincristine 18 mg was intravenously administrated for chemotherapy for lymphoma from 1 July to 4 July 2022. On 4 July, voriconazole C_min_ was 0.1 mg L^−1^ and still below the therapeutic index. His chest CT scan indicated progressive pulmonary aspergillosis with a nodule in the right upper lobe and a thick-walled cavity inside the nodule. *Aspergillus fumigatus* could be detected in bronchoalveolar lavage fluid (BALF) and blood by mNGS. Double maintenance dose of voriconazole (400 mg q12 h) was given on 5 July 2022, and voriconazole C_min_ of 1.7 mg L^−1^ (July 8th) achieved the therapeutic range. In addition, this patient carrying the CYP2C19*1*2 genotype [*2: 681G>A, rs4244285; *3: 636G>A; rs4986893; *17: −806C>T; rs12248560; genotyping adopted using the Sanger DNA sequencing method with an ABI3730xl-full automatic sequencing instrument (ABI Co.)] was an intermediate metabolizer of voriconazole. The interaction between dexamethasone and voriconazole was recognized after checking the other medications of this patient. In addition, the total score of the Drug Interaction Probability Scale (DIPS) was 10 (>8: highly probable) (Horn et al., 2007). Voriconazole C_min_ increased to 2.6 mg L^−1^ on 11 July 2022. Voriconazole dose was adjusted to 300 mg q12 h on 12 July, and voriconazole C_min_ was 2.0 mg L^−1^ on 18 July. *Aspergillus fumigatus* still could be detected in BALF by mNGS. A nodule was formed in the apex of the right lung, with a small cavity inside it, as shown by CT scan (18 July 2022). The amphotericin B cholesteryl sulfate complex with dexamethasone 2.5 mg was added on 21 July 2022. Voriconazole C_min_ was monitored, and its value was 2.0 mg L^−1^ on 27 July. The patient received the second chemotherapy regimen for lymphoma treatment consisting of dexamethasone 10 mg, etoposide 0.5 mg, and vincristine 18 mg from 1 August to 4 August. Voriconazole C_min_ was 1 mg L^−1^ on 3 August. Voriconazole dose was adjusted to 400 mg q12 h, and voriconazole C_min_ increased to 1.8 mg L^−1^ on 8 August. Then, voriconazole dose was reduced to 300 mg q12 h on 12 August. The chest CT scan indicated that symptoms of pulmonary aspergillosis improved on 16 August 2022. Voriconazole C_min_ was 1.9 mg L^−1^ (August 18th) and effective against aspergillus infection.

## Discussion

### Subtherapeutic voriconazole C_min_ associated with *CYP2C19* genotypes and dexamethasone dose

To our knowledge, this is the first report of a dose-dependent interaction between voriconazole and dexamethasone in the patient carrying the CYP2C19*1*2 genotype (IM). The co-administration of dexamethasone increased voriconazole clearance with low exposure. Voriconazole C_min_ was 0.1 mg L^−1^ (dose: 5.7 mg kg^−1^ day^−1^, 400 mg day^−1^; C_min_/dose (C/D) ratio: 0.018), when 20 mg of dexamethasone was co-administrated on 4 July 2022. Progressive pulmonary aspergillosis, indicated by CT scan and *Aspergillus fumigatus* in BALF, suggested unsatisfactory voriconazole therapy. Voriconazole C_min_ was 2 mg L^−1^ (dose: 8.6 mg kg^−1^ day^−1^, 600 mg day^−1^; C/D ratio: 0.23) when 2.5 mg of dexamethasone was used on 27 July. Then, 12.5 mg of dexamethasone was given from 1 August to 4 August. Voriconazole C_min_ was 1 mg L^−1^ (dose: 5.7 mg kg^−1^day^−1^, 400 mg day^−1^; C/D ratio: 0.18) on 3 August. Subsequent C_min_ values were 1.8 mg L^−1^ (dose: 11.4 mg kg^−1^day^−1^, 800 mg day^−1^; C/D ratio: 0.16) and 1.9 mg L^−1^ (dose: 8.6 mg kg^−1^day^−1^, 600 mg day^−1^; C/D ratio: 0.22) on 8 August and 18 August, respectively (dexamethasone: 2.5 mg). This patient received effective voriconazole therapy, combined with the amphotericin B cholesteryl sulfate complex, and showed improvement in pulmonary aspergillosis.

Reduced voriconazole C_min_ was due to increase in clearance, attributing to the co-administration of glucocorticoids (dexamethasone and methylprednisolone). Dexamethasone had a greater effect on voriconazole exposure compared to methylprednisolone (Dolton et al., 2012; Jia et al., 2021). Voriconazole C_min_ is a good measure of drug exposure, recommended as the pharmacokinetic (PK)/pharmacodynamic parameter for regular therapeutic drug monitoring (TDM) in the clinic (Takesue et al., 2022), because it has a linear relationship with the voriconazole area under the concentration–time curve (AUC_0–12h_) (Hope, 2012). However, the results of this interaction in different research studies are inconsistent (Gautier-Veyret et al., 2015; Blanco-Dorado et al., 2020). It may be related with difference in the distribution of *CYP2C19* genotypes and glucocorticoid dose employed among the studied population.

### CYP3A4 is the alternative pathway for voriconazole clearance in the IM and a poor metabolizer of CYP2C19

Voriconazole is primarily metabolized in the liver by CYP2C19 and CYP3A4 enzymes. Difference in the *CYP2C19* gene between individuals can greatly affect voriconazole metabolism. The *2 and *3 alleles were loss-of-function variations. IM with one such variant had significantly lower enzyme activity than normal metabolizer (NM, *1*1) (Li et al., 2023). In the absence of functional CYP2C19, CYP3A4 became the important alternative pathway for voriconazole clearance (Murayama et al., 2007). In CYP2C19 poor metabolizer (PM) liver microsomes, inhibition of voriconazole metabolism by ketoconazole (a specific CYP3A4 inhibitor) was most potent (Hyland et al., 2003). A PK study of healthy participants (Mikus et al., 2006) also showed that co-administration of ritonavir (a potent CYP3A4 inhibitor) led to lower voriconazole clearances in CYP2C19 IM (*1*2) and PM (*2*2), compared with NM. It is inferred that high dose of dexamethasone might affect voriconazole clearance much more in IM and PM of CYP2C19.

### Dose-dependent dexamethasone–voriconazole interaction

The extent of voriconazole–dexamethasone interaction seems to depend on the dexamethasone dose. The induction of CYP450 enzymes, particularly CYP3A4, by dexamethasone is dose-dependent (Bourdin et al., 2023). Co-administration of dexamethasone with high and moderate doses (20 and 12.5 mg) seemed to impact voriconazole C_min._ Consistently, CYP3A4 induction was, respectively, >50%–<80%, >20–<50%, and <20% at the dexamethasone dose of >16 mg (high dose), >1.5–16 mg (moderate dose), and ≤1.5 mg (low dose) (Jacobs et al., 2022). Taghvaye et al. (2019) reported the significant interaction between iv voriconazole and iv dexamethasone (8 mg q12 h), leading to failure of antifungal treatment, in a 32-year-old woman with acute lymphoblastic leukemia. Wallace et al. (2016) also found the clinical relevant drug interaction between iv voriconazole and oral dexamethasone (4 mg q8 h) in an 84-year-old male patient. In our case, subtherapeutic C_min_ of voriconazole (0.2 mg L^−1^) on 28 June and subsequent detection of *Aspergillus fumigatus* in sputum on 29 June indicated ineffective voriconazole therapy, associated with the treatment of both dexamethasone and methylprednisolone from 26 May to 27 June. The used methylprednisolone could enhance the CYP induction of high-dose dexamethasone (20 mg, from 1 July to 4 July), related with the 0.1 mg L^−1^ of voriconazole C_min_ on 4 July.

A multicenter study about the PK and TDM of voriconazole (Dolton et al., 2012) showed that co-administration of dexamethasone and methylprednisolone reduced voriconazole concentrations to a greater extent than prednisone or prednisolone, associated with their potency of binding to glucocorticoid receptors (GRs). Glucocorticoids induce CYP450s, including CYP2C19 and CYP3A4, through binding to GR. In addition to that, dexamethasone strongly induces CYP3A4 via direct activation of pregnane X receptor (PXR). A recent retrospective observational study of 231 patients with 918 voriconazole C_min_ (Jia et al., 2021) confirmed that the co-administration of glucocorticoids (dexamethasone, prednisone, prednisolone, and methylprednisolone) reduced the voriconazole C_min_/dose, among which dexamethasone caused the lowest median of the voriconazole C_min_/dose ratio. However, they did not analyze the effect of glucocorticoid dose on voriconazole. The study of immunocompromised patients (n = 38) (Imataki et al., 2018) found that voriconazole concentrations were significantly decreased in corticosteroid users compared to the non-user (*p* = .013) (the median doses of corticosteroids: 89.8 mg/d, equivalent to dexamethasone 13.47 mg). Blanco-Dorado et al. (2020) (the median doses of corticosteroids: 50 mg/d, equivalent to dexamethasone 7.5 mg) and Gautier-Veyret et al. (2015) (no data about the dose of glucocorticoids) showed no interaction between glucocorticoids and voriconazole. The inconsistent results could be explained by the heterogeneity of the type and dose of the glucocorticoids, in line with that reported in our case.

### Factors increasing the risk of clinical significant interaction

In addition, voriconazole has a long-lasting and potent inhibitory effect on CYP3A activity (Huang et al., 2021). Dexamethasone and methylprednisolone are primarily metabolized by CYP3A4 (Blanco-Dorado et al., 2020; Bourdin et al., 2023). The maximum plasma concentration (C_max_) and the AUC from 0 h to infinity (AUC_0→inf_) of dexamethasone could be increased by 2.44-fold and 2.60-fold, respectively, when combined with voriconazole. For methylprednisolone, C_max_ and AUC_0→inf_ were increased by 1.56-fold and 2.23-fold, respectively (Li et al., 2018).Thus, their increased exposure in combination with voriconazole may enhance the interaction. Multiple daily high doses (>16 mg) of dexamethasone increased the risk of clinical significant interactions. High-dose dexamethasone is widely administrated in the treatment of hematological malignancies (Jacobs et al., 2022). Furthermore, moderate-dose dexamethasone (>1.5–16 mg) with long treatment course (>10 days) had a considerable risk of interactions and commonly used in the treatment of palliative care and cerebral edema.

## Conclusion

TDM of voriconazole is necessary in patients receiving >16 mg dexamethasone with multiple daily doses, particularly those with the impaired activity of CYP2C19. Further studies regarding the interaction between glucocorticoids, especially dexamethasone and voriconazole, are warranted to prevent the clinical relevant interactions for effective antifungal therapy.

## Data Availability

The original contributions presented in the study are included in the article/Supplementary Material; further inquiries can be directed to the corresponding author.

## Abbreviations

C/D, C_min_/dose ratio; DXM, dexamethasone; IM, intermediate metabolizer; NHL, non-Hodgkin’s lymphoma; VRZ, voriconazole.
