# Supplementary material for: Case report: dose-dependent interaction between dexamethasone and voriconazole in severely ill patients with non-Hodgkin’s lymphoma being treated for invasive pulmonary aspergillosis
Source: Front Pharmacol. 2024 Jun 27;15:1403966. doi: 10.3389/fphar.2024.1403966 (PMC11236688; doi:10.3389/fphar.2024.1403966)
Supplement: Supplementary file 1 [file DataSheet1.DOCX]

**Supplementary data of**

**Case Report: Dose-dependent interaction between dexamethasone and voriconazole in a severely ill patients with non-Hodgkin's lymphoma be treating for invasive pulmonary aspergillosis**

**Details of determination of voriconazole concentrations**

Separations of voriconazole with its deuterated internal standard (voriconazole-d3) were performed on a Luna Omega C18 analytical column (50 × 2.1 mm, 1.6 μm, 100 Å, Phenomenex, Torrance, CA, USA) using a column temperature of 60°C. The plasma was eluted with mobile phase A consisting of 1 mM ammonium acetate in ultrapure water containing 0.1% (v/v) formic acid and mobile phase B consisting of acetonitrile/water (95:5, v/v) with 1 mM ammonium acetate and 0.1% (v/v) formic acid. The mobile phase had a flow rate of 0.6 mL/min with gradient elution. The gradient elution program was as follow: 0-0.8 min, 10% B; 0.8-2.0 min, 10-95% B; 2.0-2.8 min, 95% B; 2.8-2.9 min, 95-10% B; and 2.9-4.0 min, 10% B. Electrospray ionization mass spectrometry was performed in multiple reaction monitoring modes with the target ions m/z 350.1→281.2 (voriconazole) and m/z 353.2→284.2 (voriconazole-d3). The lower quantification limit was 0.1 mg/L. The linearity range of the voriconazole standard curve was 0.1-40 mg/L. The intra- and inter-day imprecision of voriconazole determination in spiked quality control samples were 1.7-5.7% and 5.3-6.9%, respectively.
